# Progression of the epidemiological transition in a rural South African setting: findings from population surveillance in Agincourt, 1993–2013

**DOI:** 10.1186/s12889-017-4312-x

**Published:** 2017-05-10

**Authors:** Chodziwadziwa W. Kabudula, Brian Houle, Mark A. Collinson, Kathleen Kahn, Francesc Xavier Gómez-Olivé, Samuel J. Clark, Stephen Tollman

**Affiliations:** 1 MRC/Wits Rural Public Health and Health Transitions Research Unit (Agincourt), School of Public Health, Faculty of Health Sciences, University of the Witwatersrand, Johannesburg, South Africa; 2 Department of Population Health, London School of Hygiene and Tropical Medicine, London, UK; 3 School of Demography, The Australian National University, Canberra, Australia; 4 CU Population Center, Institute of Behavioral Science, University of Colorado at Boulder, Boulder, CO USA; 5 INDEPTH Network, Accra, Ghana; 6 Umeå Centre for Global Health Research, Division of Epidemiology and Global Health, Department of Public Health and Clinical Medicine, Umeå University, Umeå, Sweden; 7 Department of Sociology, The Ohio State University, Columbus, OH USA

**Keywords:** South Africa, Agincourt, Rural, Mortality, HIV/Aids, Cause composition, Verbal autopsy, InterVA, Non-communicable diseases, epidemiological transition

## Abstract

**Background:**

Virtually all low- and middle-income countries are undergoing an epidemiological transition whose progression is more varied than experienced in high-income countries. Observed changes in mortality and disease patterns reveal that the transition in most low- and middle-income countries is characterized by reversals, partial changes and the simultaneous occurrence of different types of diseases of varying magnitude. Localized characterization of this shifting burden, frequently lacking, is essential to guide decentralised health and social systems on the effective targeting of limited resources. Based on a rigorous compilation of mortality data over two decades, this paper provides a comprehensive assessment of the epidemiological transition in a rural South African population.

**Methods:**

We estimate overall and cause-specific hazards of death as functions of sex, age and time period from mortality data from the Agincourt Health and socio-Demographic Surveillance System and conduct statistical tests of changes and differentials to assess the progression of the epidemiological transition over the period 1993–2013.

**Results:**

From the early 1990s until 2007 the population experienced a reversal in its epidemiological transition, driven mostly by increased HIV/AIDS and TB related mortality. In recent years, the transition is following a positive trajectory as a result of declining HIV/AIDS and TB related mortality. However, in most age groups the cause of death distribution is yet to reach the levels it occupied in the early 1990s. The transition is also characterized by persistent gender differences with more rapid positive progression in females than males.

**Conclusions:**

This typical rural South African population is experiencing a protracted epidemiological transition. The intersection and interaction of HIV/AIDS and antiretroviral treatment, non-communicable disease risk factors and complex social and behavioral changes will impact on continued progress in reducing preventable mortality and improving health across the life course. Integrated healthcare planning and program delivery is required to improve access and adherence for HIV and non-communicable disease treatment. These findings from a local, rural setting over an extended period contribute to the evidence needed to inform further refinement and advancement of epidemiological transition theory.

**Electronic supplementary material:**

The online version of this article (doi:10.1186/s12889-017-4312-x) contains supplementary material, which is available to authorized users.

## Background

Over time, mortality and disease patterns in human populations transition from very high and fluctuating mortality concentrated at younger ages and largely caused by infectious diseases and nutritional deficiencies to relatively stable low mortality concentrated at older ages and largely caused by non-communicable diseases and injuries – the ‘epidemiological transition’ [1]. High-income countries experienced this transition in an orderly way along a unidirectional path during the first half of the twentieth century [1]. The first phase of the transition was characterized by high, fluctuating mortality dominated by epidemics of infectious diseases, famines and wars. Thereafter, mortality rates declined progressively and degenerative diseases started to replace infectious diseases as the major causes of morbidity and death. Finally, in later stages of the transition, non-communicable diseases such as cardiovascular diseases, diabetes and cancers, and accidents became the main causes of death, and mortality rates eventually stabilized at relatively low levels [1–3]. In low- and middle-income countries the epidemiological transition is still underway and its progress is more varied compared to the experience of high-income countries. Observed changes in mortality and disease patterns in most low- and middle-income countries including those in sub-Saharan Africa reveal transitions that are characterized by reversals, partial changes and simultaneous occurrence of different types of diseases [4–14].

For a long time in South Africa there was a steady decrease in the level of overall mortality. This trend was reversed by the HIV/AIDS epidemic that dramatically increased overall mortality from the mid-1990s to the mid-2000s [6, 15–20]. In recent years, the availability and use of antiretroviral treatment is reducing HIV/AIDS-related mortality and life expectancy is rising [18, 21, 22]. At the same time, modernization, economic and social development over the past two decades have resulted in the adoption of lifestyle practices that expose South Africans to a variety of risk factors for non-communicable diseases and injuries. Hence, the cause of death profile of South Africans increasingly includes non-communicable diseases, violence and injuries [18, 21–30].

As the epidemiological transition continues to unfold in South Africa, influenced by broader demographic, socioeconomic, technological, political, and cultural changes, there is ongoing need to quantify and characterize it and its implications in different sub-populations. This will reveal the history of the burden of disease affecting different ethnic and social groups and help identify and prioritize the interventions with potential for the greatest effect now and in the near future. This need was highlighted by the Global Burden of Disease study [31], which characterized the extent of regional heterogeneity in the trajectories of the epidemiological transition and called for greater availability and understanding of local, national, and regional data. Characterizing the shifting burden of mortality over time is critical in areas without reliable data – particularly rural settings where a greater evidence base can inform the targeting of limited resources and identify rural-urban differences and disparities.

Using mortality and cause of death data from the Agincourt Health and Socio-Demographic Surveillance System (HDSS), this article provides a comprehensive assessment of the epidemiological transition in a rural population in northeast South Africa over the period 1993–2013. This period spans major socio-political changes, the start of the HIV/AIDS epidemic and availability of antiretroviral treatment. In the article we significantly improve, update and extend measures of the trends in mortality and cause of death profiles for the Agincourt study population that have appeared earlier [6, 18, 28]. Importantly, unlike the previous work we operationalize the epidemiological transition using a statistical framework that allows us to characterize its progress relating overall mortality levels to changes in the cause composition and conduct statistical tests of changes and differentials. The longitudinal empirical evidence from this study adds a further rural South African dimension to the sparse literature on the current experience of the epidemiological transition across diverse places and contexts in low- and middle-income settings.

## Methods

### Data

We use mortality and cause of death data collected from 1993 to 2013 as part of annual updates of vital events conducted using the Agincourt HDSS in a population occupying 27 villages in rural northeast South Africa [32, 33]. The population is largely Shangaan (Tsonga)-speaking. Former Mozambican refugees, who arrived in the study area in the early to mid-1980s in the course and aftermath of civil war, and their descendants, make up about 30% of the population. The population has been under epidemiological and demographic surveillance since 1992 and vital events were updated at approximately 15- to 18-month intervals between 1993 and 1999, and annually since 1999.

Although the population has limited access to infrastructure and public sector services, it has experienced substantial socioeconomic changes over the years. As documented in our earlier study [34], the proportion of households that own assets associated with greater modern wealth has increased substantially over time. For example, the proportion of households with dwellings constructed with either brick or cement walls increased from 76% in 2001 to 98% in 2013; and the prevalence of tiles as roof and floor materials of dwellings increased respectively from 3% and 0.5% in 2001 to 15% and 14% in 2013. In addition, the use of electricity for lighting and cooking respectively increased from 69% and 4% of households in 2001 to 96% and 50% of households in 2013. Other notable increases are in the proportion of households owning stove, fridge, cellphone and car respectively from 41%, 40%, 37% and 14% in 2001 to 85%, 86%, 98% and 20% in 2013.

For individuals identified as having died between the annual surveillance update rounds, verbal autopsy (VA) interviews were conducted with their caregivers to elicit signs and symptoms of the illness or injury prior to their death. The interviews were conducted one to 11 months after death using a locally validated, local-language VA instrument [33, 35].

Given the rigorous processes involved in the collection, quality assurance and processing of HDSS data [14, 36], the data from the Agincourt HDSS population is one of the rare high-quality and methodologically consistent longitudinal health and demographic dataset for populations in resource-poor low- and middle-income settings. The available mortality and cause of death information by age and sex over an extended period provides a unique opportunity for assessing how populations in low- and middle-income settings, including those in rural sub-Saharan Africa are currently experiencing the epidemiological transition.

### Assigning causes of death

We use the InterVA-4 probabilistic model (version 4.03) to assign probable causes of death to every death with a complete VA interview. For each death, the InterVA-4 model assigns up to three likely causes of death with associated likelihoods [37]. An indeterminate cause of death is assigned when the VA information is inadequate for the model to arrive at any cause of death. We opted for InterVA-4 as opposed to physician-coded causes of death because the InterVA-4 model assigns causes of death in a standardized, automated manner that is much quicker and more consistent than the former (particularly for assessing changes over time and across settings). Additionally, causes of death derived from InterVA-4 have been found to not substantially differ from those generated by physician coding [38].

### Statistical analysis

#### Trends in mortality and causes of death

Similar to some earlier studies [28, 39], we use discrete-time event history analysis (DTEH) [40] to estimate overall and cause-specific annual hazards of death as functions of sex, age and time period. The *annual hazard* of dying is the probability of dying during a one-year interval starting on a particular date experienced by living individuals, conditional on their state at the beginning of the interval. An individual’s continuously evolving *state* is described by the combination of values taken by both constant and time-varying variables, for this study, sex, age and time period.

One of the basic requirements of DTEH is the splitting of each individual’s survival history into a set of discrete person years [40]. We create a person-year file that contains one record for each full year lived by each individual in the study population. For example, individuals who died after one year of surveillance contribute one person-year each while those who died after five years of surveillance contribute five person-years. Only *completely observed* person-years are included in the data set except when an individual dies before completing a person-year time unit. Survival histories are truncated for individuals who were alive at the beginning or end of the study and for those who migrated in/out during the study.

After constructing the person-year file we estimate the annual hazards of dying using logistic regression models [40–44]. Binary logistic regression models are used for estimates of the risk of dying from all possible causes, and multinomial logistic regression models are used to obtain estimates of the risk of dying from causes in broad cause of death categories. Using the estimated annual hazards of death, we construct standard life tables to derive life expectancies at birth and adult mortality rates (the probability of dying between ages 15 and 60 for those who survive to age 15 if subjected to age-specific mortality rates between those ages for the specified calendar year).

In order to contextualize the dynamics of the HIV epidemic and the availability of antiretroviral treatment over time, the years of the study are divided into the following time periods: 1993–1997, 1998–2000, 2001–2003, 2004–2007, 2008–2010 and 2011–2013. We also categorize age into the following commonly used age groups: 0–4, 5–14, 15–49, 50–64 and 65+. For the cause-specific analyses, the most likely causes of death generated by the InterVA-4 model except indeterminate are categorized into four broad groups: (1) HIV/AIDS and TB; (2) other communicable, maternal, perinatal, and nutritional diseases (excluding HIV/AIDS and TB); (3) non-communicable diseases; and (4) injuries, consistent with the burden of disease classification system in South Africa [23].

#### Changes in mortality and cause of death patterns

Following a common, standard approach to analyzing changes in mortality and cause of death patterns, we divide the most likely causes of death generated by the InterVA-4 model into three broad cause groups that can be compared with existing publications: Group I (communicable diseases, maternal, and perinatal conditions and nutritional deficiencies), Group II (non- communicable diseases), and Group III (accidents and injuries) [45, 46]. The proportion of deaths attributed to each cause group ranges from 0 to 1 and the set of proportions for all of the cause groups sums to 1 after excluding indeterminate causes. We follow Salomon and Murray [46] to relate the distribution of deaths among cause groups to the overall level of mortality. We fit estimates of age and cause-specific mortality fractions to a set of regression equations of the following form

1 $$ {Y}_{i1}={\beta}_0+{\beta}_1 \ln \left({M}_i\right)+{\varepsilon}_{i1},\mathrm{and} $$

2 $$ {Y}_{i2}={\gamma}_0+{\gamma}_1 \ln \left({M}_i\right)+{\varepsilon}_{i2}. $$

where *i* indexes age; *Y*_*i*1_ and *Y*_*i*2_ are the log ratios of the cause-specific fractions for Group II causes (P_2_) and Group III causes (P_3_) relative to the cause-specific fraction for Group I causes (P_1_): ${Y}_{i1}= \ln \left(\frac{P_2}{P_1}\right)$ and ${Y}_{i2}= \ln \left(\frac{P_3}{P_1}\right)$; *M*_*i*_ is the all-cause mortality rate; *β*_0_ and *γ*_0_ are constant terms and *ε*_*i*1_ and *ε*_*i*2_ are error terms. The coefficients are estimated using *seemingly unrelated regression* models*,* separately for each sex and age group. These models provide efficient means of jointly obtaining estimates from a set of equations each with its own error term that may be correlated with the error terms of other equations. As in Salomon and Murray [46] we compute predicted values for Y_1_ and Y_2_ for each observation in the dataset. Those predicted values are transformed into predicted proportions for each cause group using the multivariate logistic transformation:

$$ {P}_j=\frac{ \exp \left({Y}_j\right)}{1+\sum_{j=1}^{J-1}\  \exp \left({Y}_j\right)} $$

where *J* = 3 and *P*_3_ is 1 − *P*_1_−*P*_2_.

### Software

All analyses have been conducted using Stata version 14.1 (Stata Corp., College Station, USA).

## Results

Over the period 1993–2013 the Agincourt HDSS recorded a total of 13,472 deaths in 1,604,085 person-years of follow-up. Table 1 presents the person-years and number of deaths grouped by time period and cause of death categories. VA interviews were available for 92% of the deaths. VA interviews were not conducted for the other 8% of the deaths mainly due to failure to contact suitable respondents. The InterVA-4 model assigned undetermined cause of death to 6.2% of the deaths with VA interviews.

**Table 1:** Person years and number of deaths by time period and cause of death categories, Agincourt, South Africa, 1993–2013

Sex	Indicator	1993–1997	1998–2000	2001–2003	2004–2007	2008–2010	2011–2013
Female	Person years	174,518	108,599	110,608	155,062	138,883	145,799
	Number of Deaths						
	Total	773	677	1019	1651	1229	1096
	HIV/AIDS & TB	200	226	505	825	476	286
	Other Communicable	139	109	126	219	220	237
	Non Communicable	248	203	238	391	424	452
	Injuries	47	30	38	46	29	31
	Indeterminate	52	46	44	93	48	41
	VA interview not done	87	63	68	77	32	49
Male	Person years	161,119	101,311	102,972	143,188	127,695	134,331
	Number of Deaths						
	Total	900	708	1115	1833	1363	1108
	HIV/AIDS & TB	243	256	480	755	528	300
	Other Communicable	120	97	126	236	271	221
	Non Communicable	234	158	230	420	337	352
	Injuries	130	79	119	160	102	127
	Indeterminate	44	20	40	74	49	46
	VA interview not done	129	98	120	188	76	62

### Trends in mortality and cause of death

**Fig. 1:**
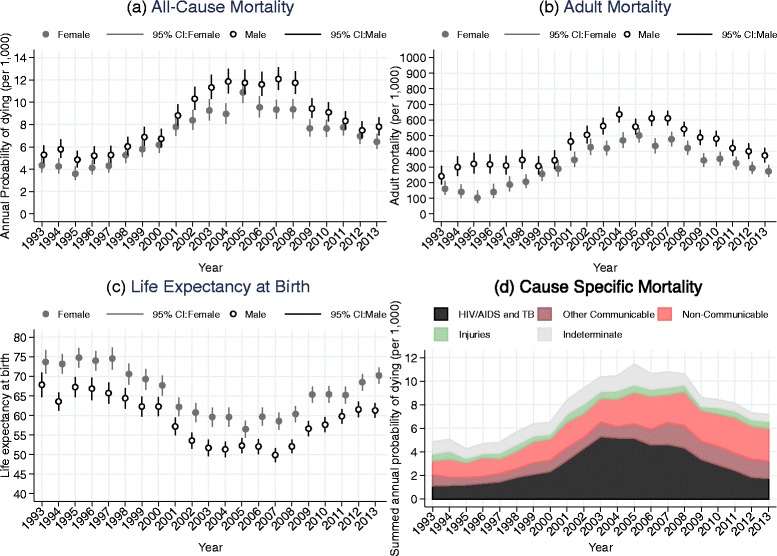
Trends in selected mortality and cause of death indicators, Agincourt, South Africa, 1993–2013. **a** All-Cause Mortality. **b** Adult Mortality. **c** Life Expectancy at Birth. **d** Cause Specific Mortality

Table 2 presents summed annual probabilities of dying from all causes for all age groups (per 1000), adult (ages 15–64) mortality rates (per 1000) and life expectancies at birth. These estimates describe trends in all-cause mortality and are also shown in Fig. 1 panels (a), (b) and (c).

**Table 2:** Trends in selected mortality indicators, Agincourt, South Africa, 1993–2013

Sex	Year	Annual probability of dying (95% CI)	Adult mortality rate (95% CI)	Life Expectancy at birth (95% CI)
Female	1993	4.5 (3.8,5.2)	200.3 (159.7249.6)	73.7 (70.7,76.7)
	1994	4.5 (3.8,5.2)	171.8 (134.2218.6)	73.2 (70.7,75.6)
	1995	3.8 (3.2,4.5)	127.6 (93.6172.8)	74.8 (72.3,77.2)
	1996	4.3 (3.6,5.0)	166.7 (127.6216.4)	74 (71.5,76.5)
	1997	4.5 (3.9,5.2)	222.7 (180.1273.6)	74.6 (71.7,77.4)
	1998	5.5 (4.8,6.3)	242 (202.2288.2)	70.6 (67.9,73.3)
	1999	6.1 (5.3,6.9)	296.5 (254.2344.1)	69.3 (66.6,71.9)
	2000	6.4 (5.6,7.2)	322.5 (275.3375.4)	67.7 (65.1,70.2)
	2001	8.1 (7.3,9.1)	392 (347,440.6)	62.2 (59.8,64.5)
	2002	8.7 (7.9,9.7)	469.2 (421.8519.3)	60.8 (58.3,63.2)
	2003	9.6 (8.6,10.6)	463.5 (420.2509)	59.6 (57.2,62.1)
	2004	9.4 (8.5,10.4)	503.3 (457.8550.8)	59.6 (57.2,62)
	2005	11.3 (10.3,12.4)	536.4 (493.6580.5)	56.5 (54.3,58.7)
	2006	9.9 (9.0,11.0)	476.2 (432,522.5)	59.7 (57.3,62)
	2007	10.2 (9.3,11.2)	517.9 (474.6562.7)	58.6 (56.3,60.8)
	2008	9.7 (8.8,10.6)	464.3 (422.8507.9)	60.4 (58.3,62.4)
	2009	8.0 (7.3,8.9)	378.6 (338.3421.9)	65.3 (63.2,67.4)
	2010	8.0 (7.2,8.8)	377.1 (337,420.3)	65.4 (63.3,67.5)
	2011	7.9 (7.2,8.8)	359 (321.8399.2)	65.2 (63.1,67.3)
	2012	7.2 (6.5,8)	317.2 (280.9357)	68.5 (66.4,70.5)
	2013	6.7 (6,7.4)	294.9 (258.2335.5)	70.2 (68.2,72.3)
Male	1993	5.4 (4.7,6.3)	281.8 (229.5343.3)	67.8 (64.7,70.9)
	1994	6 (5.2,6.9)	324.4 (267.8389.4)	63.6 (61.3,65.8)
	1995	5 (4.3,5.8)	343.5 (284.6410.6)	67.2 (64.7,69.8)
	1996	5.3 (4.6,6.2)	337.8 (282.7400.3)	66.9 (64,69.7)
	1997	5.4 (4.7,6.3)	331 (277.1392.2)	65.7 (63.1,68.4)
	1998	6.2 (5.4,7.1)	373 (317.6434.7)	64.4 (61.7,67)
	1999	7.1 (6.3,8.1)	358.5 (306.6416.1)	62.3 (59.8,64.8)
	2000	7 (6.2,8)	382 (328.9440.4)	62.3 (59.8,64.7)
	2001	9.1 (8.1,10.1)	499.3 (446.5554.7)	57.2 (55,59.4)
	2002	10.5 (9.5,11.7)	550.4 (499.3603)	53.6 (51.5,55.7)
	2003	11.8 (10.7,12.9)	601.3 (552.8650.3)	51.7 (49.6,53.8)
	2004	12 (10.9,13.2)	662.6 (615.9708.8)	51.3 (49.3,53.3)
	2005	12.1 (11,13.3)	588.5 (541.2636.4)	52.3 (50.4,54.1)
	2006	11.9 (10.9,13.1)	637.1 (591.5682.6)	52.1 (50.1,54)
	2007	13.2 (12.1,14.4)	650 (607.3692.4)	49.9 (48.1,51.7)
	2008	12.1 (11.1,13.2)	581.7 (539.4624.6)	52.1 (50.3,53.8)
	2009	9.8 (9,10.8)	517.4 (473.2563.2)	56.6 (54.7,58.5)
	2010	9.3 (8.5,10.3)	511.9 (466.4559.1)	57.6 (55.7,59.5)
	2011	8.6 (7.8,9.5)	445.4 (398.7495.1)	59.8 (58,61.6)
	2012	7.7 (6.9,8.5)	426.1 (380.2475)	61.5 (59.6,63.5)
	2013	7.9 (7.2,8.8)	399.7 (355.4447.3)	61.3 (59.4,63.2)

Adult mortality rate is the probability of dying between ages 15 and 59

The annual probability of dying from all causes for all ages was about 5.4 and 4.5 per 1000 person years in 1993 for males and females, respectively. Those started to increase rapidly around 1997 for both males and females and reached a peak level around 2007 of 13.2 per 1000 person years for males and 11.3 per 1000 person years around 2005 for females, before starting to decline in more recent years. By 2013, the annual probability of dying from all causes had reduced from peak levels to 7.9 per 1000 person years for males and 6.7 per 1000 person years for females. At the peak overall mortality for both sexes had more than doubled to about 2.5 times its starting value.

Adult mortality rates exhibit a similar pattern. From a base of 281.8 and 200.3 per 1000 person years in 1993 for males and females, adult mortality rose to 650 per 1000 person years around 2007 for males and 536 per 1000 person years around 2005 for females, before starting to decline. By 2013 adult mortality rates had reduced from the peak levels to 399.7 per 1000 person years for males and 294.9 per 1000 person years for females.

Trends in life expectancy at birth reflect trends in all-age and adult mortality for both sexes. For females, life expectancy at birth dropped from about 74 years in 1993 to about 57 years in 2005 (a loss of 17 years) and returned to around 70 years by 2013. For males, life expectancy at birth dropped from about 68 years in 1993 to about 50 years in 2007 (a loss of 18 years) and increased to around 61 years by 2013.

**Fig. 2:**
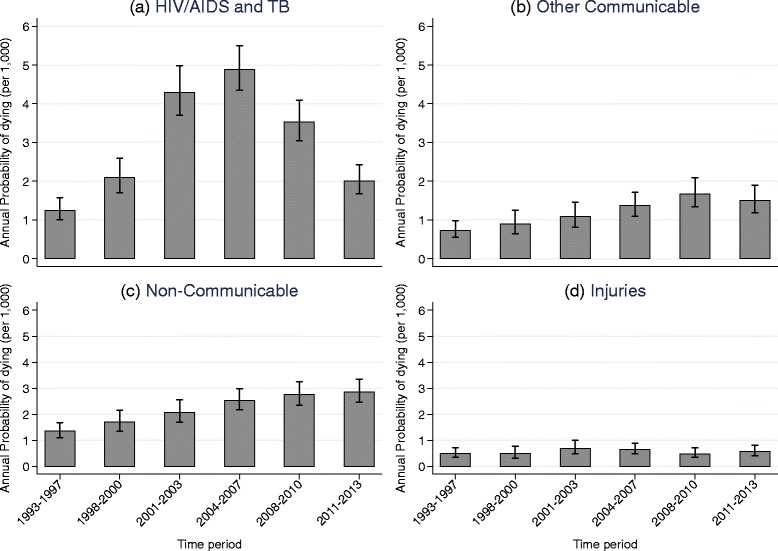
Trends in annual probability of dying by cause of death, Agincourt, South Africa, 1993–2013. **a** HIV/AIDS and TB. **b** Other Communicable. **c** Non-Communicable. **d** Injuries

Figure 1 panel (d) shows the predicted summed annual probability of dying per 1000 person years by year and cause of death, estimated using a multinomial logistic regression. The estimated probability of dying from each cause is added to that of the other causes. Hence, the cumulative area under each curve represents the probability of dying from the successively combined causes in a given year. The annual probability of dying from HIV/AIDS and TB increased dramatically after 2000, reached a peak around 2004–2005 and has been decreasing since 2007. However, the level of HIV/AIDS and TB related mortality in the more recent time is still higher than the level in 1993. Non-communicable diseases have consistently been the next largest cause of death and the probability of dying from them has increased steadily over time. The probability of dying from accidents and injuries has remained steady and low although there is a major difference between males and females. Figure 2 shows summaries of the same trends in the probabilities of dying from the different cause of death categories with the years of follow-up divided into six time periods.

**Fig. 3:**
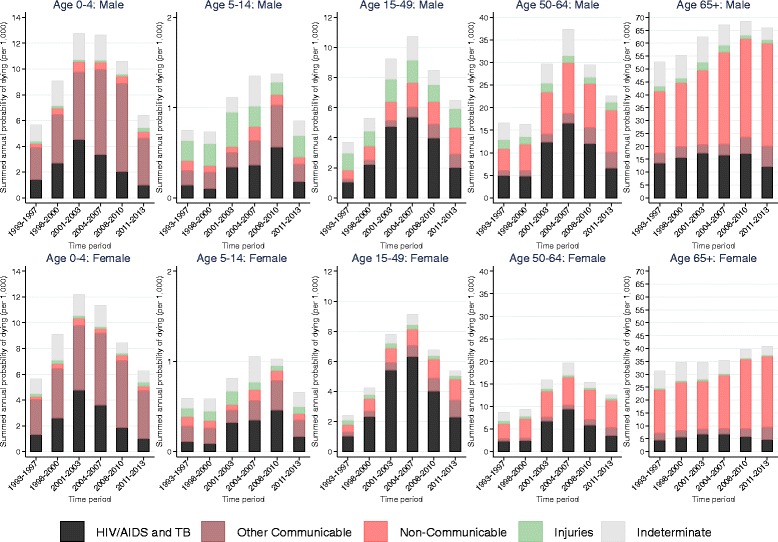
Trends in annual probability of dying by age, sex and cause of death, Agincourt, South Africa, 1993–2013

**Fig. 4:**
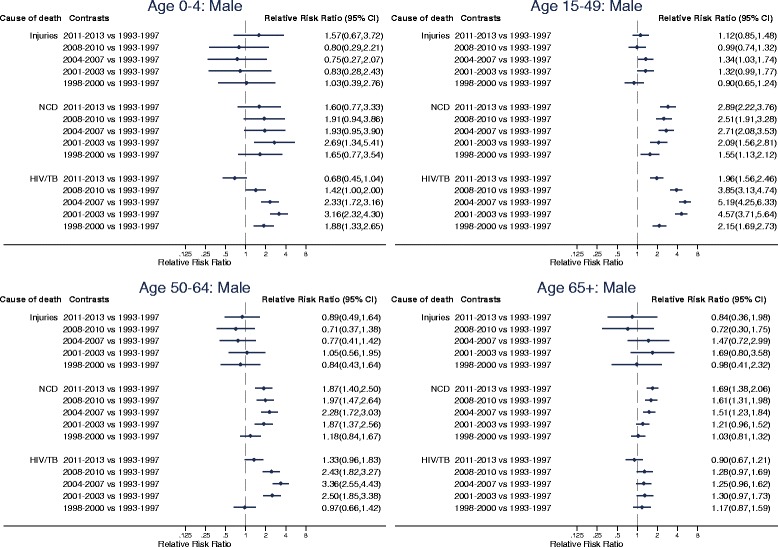
Age-specific marginal linear predictions of dying from selected causes of death in subsequent time periods relative to 1993–1997 for males, Agincourt, South Africa, 1993–2013

**Fig. 5:**
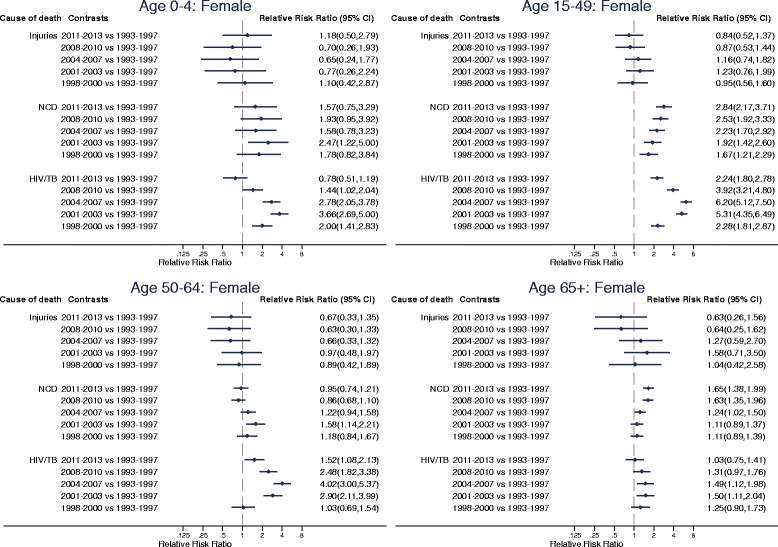
Age-specific marginal linear predictions of dying from selected causes of death in subsequent time periods relative to 1993–1997 for females, Agincourt, South Africa, 1993–2013

Estimates of the risk of dying from different causes as a function of sex, age and time period are presented in Table S1 (see Additional file 1). Trends in the probabilities of dying from each of the cause of death categories by sex, age and time period, and the age-specific marginal linear predictions of dying from selected causes in subsequent time periods relative to 1993–1997 obtained from these estimates, are displayed in Figs. 3, 4 and 5.

The vertical scales for the probabilities of dying in Fig. 3 are appreciably different between the different age categories. Throughout time and for all ages, males have higher probability of dying from all causes compared to females. In all time periods, those aged 65+ have the highest probabilities of dying from all causes followed respectively in descending order by those aged 50–64, 0–4, 15–49 and 5–14.

In the 65+ age category non-communicable diseases have been the leading cause of death for both males and females and mortality associated with them has been rising steadily. Significant increases in non-communicable disease mortality started to emerge in this age category from 2004 to 2007 (RRR (95% CI): 1.51 (1.23, 1.84), *p* < 0.001 for males and RRR (95% CI): 1.24 (1.02, 1.50), *p* = 0.0271 for females in 2004–2007 relative to 1993–1997). By 2011–2013, the risk of dying from non-communicable diseases in this age category reached 1.69 (95% CI: 1.38, 2.06, *p* < 0.001) times as large as 1993–1997 for males and 1.65 (95% CI: 1.38, 1.99, *p* < 0.001) times as large as 1993–1997 for females.

In the 50–64-age category non-communicable diseases have also been an important cause of death although the risk of dying from them over time relative to 1993–1997 has remained almost constant for both males and females. The risk of dying from non-communicable diseases relative to 1993–1997 increased to statistically significant levels in 2001–2003 (RRR (95% CI): 1.87 (1.37,2.56), *p* < 0.001) and remained constant thereafter in males and was only statistically significant in 2001–2003 (RRR (95% CI): 1.58 (1.14,2.21), *p* = 0.007) in females.

HIV/AIDS and TB have also contributed significantly to mortality in the 50–64 age category for both males and females. The probability of dying from HIV/AIDS and TB in this age category steadily increased from the late 1990s to the early 2000s and reached a peak in 2004–2007 (RRR (95% CI): 3.36 (2.55, 4.43), *p* < 0.001 in 2004–2007 relative to 1993–1997 for males; RRR (95% CI): 4.02 (3.00, 5.37), *p* < 0.001 in 2004–2007 relative to 1993–1997 for females). Since then, HIV/AIDS and TB mortality has steadily declined and has reached the baseline level for males (RRR (95% CI): 1.33 (0.96,1.83), *p* = 0.083 in 2011–2013 relative to 1993–1997) and is closer to baseline level for females (RRR (95% CI): 1.52 (1.08–2.13), *p* = 0.016 in 2011–2013 relative to 1993–1997).

In the 15–49 age category for both males and females HIV/AIDS and TB have been the leading causes of death. The probability of dying from HIV/AIDS and TB in this age category increased dramatically from the late 1990s to the early 2000s, reached a peak in 2004–2007 (RRR (95% CI): **5.19** (4.25, 6.33), *p* < 0.001 in 2004–2007 relative to 1993–1997 for males; RRR (95% CI): **6.20** (5.12, 7.50), *p* < 0.001 in 2004–2007 relative to 1993–1997 for females) and has steadily decreased since then. Notwithstanding, HIV/AIDS and TB mortality in the most recent time periods is still above what it was during the early 1990s for both males (RRR (95% CI): 1.96 (1.56,2.46), *p* < 0.001 in 2011–2013 relative to 1993–1997) and females (RRR (95% CI): 2.24 (1.80–2.78), *p* < 0.001 in 2011–2013 relative to 1993–1997). Both males and females in the 15–49 age category have also experienced steady increases in the risk of dying from non-communicable diseases over the years. By 2011–2013, the risk of dying from non-communicable diseases in this age category reached 2.89 (95% CI: 2.22, 3.76, *p* < 0.001) times as large as 1993–1997 for males and 2.84 (95% CI: 2.17, 3.71, *p* < 0.001) times as large as 1993–1997 for females.

In the 5–14 age category overall mortality increased steadily from the late 1990s, reached a peak in 2004–2007 and remained high until 2008–2010. Overall mortality in this age group only started to decline in the most recent time period following reductions in mortality from HIV/AIDS and TB. The risk of dying from HIV/AIDS and TB in this age category reached peak levels of 3.97 (95% CI: 2.07, 7.62, *p* < 0.001) times as large as 1993–1997 for males and 4.04 (95% CI: 2.09, 7.79, *p* < 0.001) times as large as 1993–1997 for females in 2008–2010 but dropped to the same level as 1993–1997 in 2011–2013 (RRR (95% CI): 1.27 (0.56, 2.86), *p* = 0.565 in 2011–2013 relative to 1993–1997 for males; RRR (95% CI): 1.45 (0.64, 3.28), *p* = 0.371 in 2011–2013 relative to 1993–1997 for females).

In the 0–4 age group mortality has almost exclusively been from HIV/AIDS and TB and other communicable diseases. Trends in the pattern of overall mortality in this age category have mirrored those of the HIV/AIDS mortality pattern. For both males and females, HIV/AIDS mortality in this age group increased steadily from the mid 1990s, reached a peak in 2001–2003 (RRR (95% CI): 3.16 (2.32, 4.30), *p* < 0.001 in 2001–2003 relative to 1993–1997 for males; RRR (95% CI): 3.66 (2.69, 5.00), *p* < 0.001 in 2001–2003 relative to 1993–1997 for females) and has steadily decreased since then. In the most recent time period the level of HIV/AIDS mortality in this age category is equivalent to the level it was during the 1993–1997 time period (RRR (95% CI): 0.68 (0.45, 1.04), *p* = 0.075 in 2011–2013 relative to 1993–1997 for males; RRR (95% CI): 0.78 (0.51, 1.19), *p* = 0.249 in 2011–2013 relative to 1993–1997 for females).

### Shifts in mortality and cause of death patterns

**Fig. 6:**
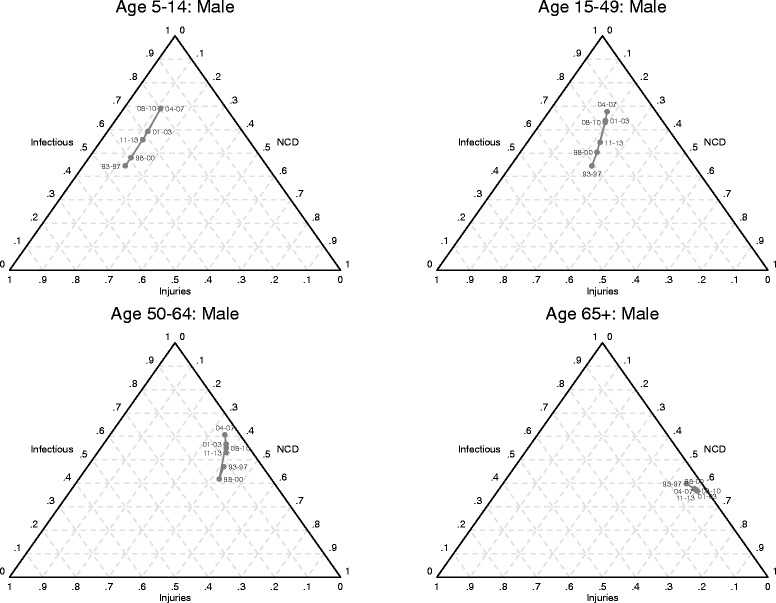
Shifts in mortality and cause of death patterns for males, Agincourt, South Africa, 1993–2013

**Fig. 7:**
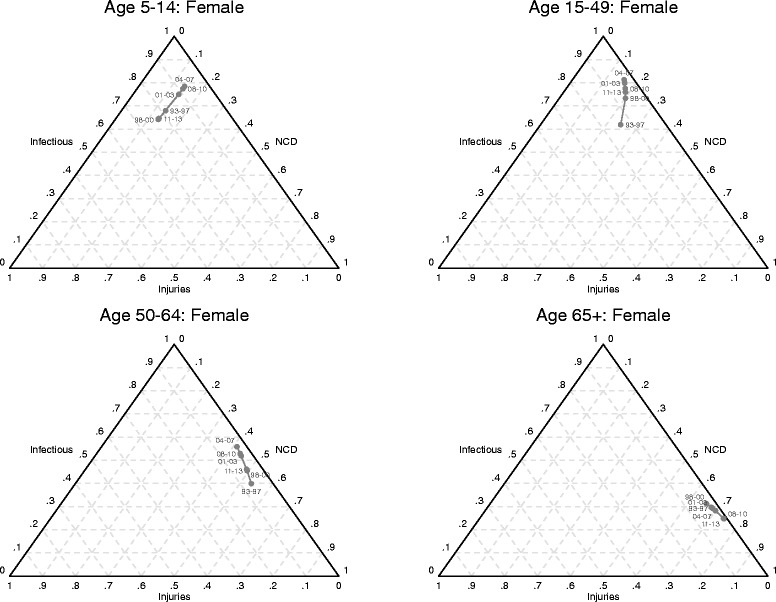
Shifts in mortality and cause of death patterns for females, Agincourt, South Africa, 1993–2013

Table 3 and Figs. 6 and 7 present results from the analysis of the progression of the epidemiological transition in the Agincourt study population for each sex, age category. Table 3 shows the estimated coefficients from the seemingly unrelated regression models. For most age groups, the coefficients for all-cause mortality are statistically significant. Figures 6 and 7 show, for males and females respectively, the actual predicted proportions of deaths attributed to each cause group using ternary diagrams. A ternary diagram is a graphical representation of three variables which sum to a constant and depicts ratios of three variables as positions in an equilateral triangle. The diagrams reveal more detail on the progression of the transition for each sex and age category. The points within each diagram are labeled with an abbreviation of the time period that omits the century, for example 93–97 for 1993–1997. Each point simultaneously represents the fraction of deaths attributed to the standard Group I (communicable diseases, maternal, and perinatal conditions and nutritional deficiencies), Group II (non- communicable diseases), and Group III (accidents and injuries) cause categories. The fraction of deaths attributed to Group I causes is represented as the perpendicular distance from the bottom of the triangle to the top vertex; the fraction to Group II causes is the perpendicular distance from the left side of the triangle to the right vertex; and the fraction to Group III is the perpendicular distance from the right side of the triangle to the left vertex. For example, the point labeled 93–97 for males aged 15–49 represents 47% of deaths from Group I, 22% deaths from Group II and 31% deaths from Group III. Diagrams for the 0–4 age group are not included because there is little movement in this age group. Thus the predictions show the direction and magnitude of changes in cause of death patterns as mortality changes.

**Table 3:** Seemingly unrelated regression estimates of log ratios of cause fractions on all-cause mortality

Sex	Coefficients for ln(P_2_/P_1_)					Coefficients for ln(P_3_/P_1_)			
	Age	ln(*M*_*i*_) (95% CI)	*p*-value	Intercept (95% CI)	*p*-value	ln(*M*_*i*_) (95% CI)	*p*-value	Intercept (95% CI)	*p*-value
Female	0–4	−0.60 (−0.91,−0.28)	< 0.001	−5.68 (−7.16,−4.21)	< 0.001	−0.51 (−1.54,0.52)	0.329	−5.77 (−10.61,−0.93)	0.019
	5–14	−0.20 (−1.97,1.58)	0.830	−3.05 (−15.71,9.62)	0.637	−1.89 (−3.78,0.01)	0.051	−15.09 (−28.62,−1.57)	0.029
	15–49	−0.49 (−1.01,0.03)	0.063	−3.92 (−6.60,−1.24)	0.004	-1.24 (−1.66,−0.82)	< 0.001	−9 (−11.17,−6.84)	< 0.001
	50–64	−0.61 (−0.97,−0.26)	0.001	−2.69 (−4.21,−1.16)	0.001	−1.15 (−1.84,−0.46)	0.001	−7.37 (−10.35,−4.39)	< 0.001
	65+	1.70 (0.50,2.91)	0.006	6.54 (2.56,10.52)	0.001	−4.06 (−6.03,−2.09)	< 0.001	−16.16 (−22.67,−9.66)	< 0.001
Male	0–4	0.01 (−0.71,0.72)	0.983	−2.56 (−5.87,0.75)	0.129	−2.9 (−3.96,−1.83)	< 0.001	−17.25 (−22.18,−12.32)	< 0.001
	5–14	−0.85 (−2.28,0.59)	0.249	−7.34 (−17.19,2.51)	0.144	−1.8 (−3.12,−0.47)	0.008	−12.98 (−22.06,−3.9)	0.005
	15–49	−0.74 (−1.08,−0.39)	< 0.001	−4.63 (−6.32,−2.94)	< 0.001	−1.13 (−1.42,−0.84)	< 0.001	−6.57 (−8.02,−5.12)	< 0.001
	50–64	−0.59 (−1.05,−0.13)	0.011	−2.50 (−4.20,−0.79)	0.004	−1.66 (−2.29,−1.03)	< 0.001	−8.01 (−10.35,−5.67)	< 0.001
	65+	0.46 (−0.36,1.28)	0.270	1.73 (−0.54,4.00)	0.136	−1.08 (−3.89,1.73)	0.451	−5.41 (−13.18,2.36)	0.172

*M*_*i*_ is the all-cause mortality rate for age category *i*; P_1_ is cause-specific fraction for Group I causes; P_2_ is cause-specific fraction for Group II causes and P_3_ is cause-specific fraction for Group III causes.

From the perspective of classical understanding of epidemiological transition, the diagrams show reversals in the progression of the transition in most age groups during the periods of rising HIV/AIDS mortality. For most age groups, the reversals peak during the 2004–2007 time period. The classic epidemiological transition is on track (conforms to standard understanding) in recent years following the widespread availability and uptake of antiretroviral treatment. However, the progression of the transition for most age categories has not yet quite recovered back to the stage it was in the early 1990s.

The diagrams also reveal the simultaneous impacts and interactions of different cause groups as the epidemiological transition progresses in the positive direction, i.e. overall mortality falling. The time trajectory of paths charting the progression of the transition varies by sex. For the older age groups, the relative importance of mortality from non-communicable diseases increases first for females and then for males, although non-communicable diseases become the dominant cause of death for both sexes as all-cause mortality falls.

For young and middle-aged adults (15–49 years), the relative contribution of mortality from injuries increases more for males compared to females as the transition progresses. For young and middle-aged adult females, the relative contribution of mortality from communicable causes is higher than for their male counterparts.

## Discussion

This paper has assessed the progress of the epidemiological transition in a rural population in South Africa undergoing profound health and social changes, using mortality and cause of death data collected over two decades through a robust health and socio-demographic surveillance system. The findings improve, update and extend published trends in mortality and cause of death profiles [6, 18, 28] by including data from more recent years that cover the widespread availability and uptake of ART. Further, the analytical approach allows for the progress of the epidemiological transition to be empirically assessed by relating overall mortality levels to changes in the cause composition over time.

The results clearly exhibit elements of the “counter” and “protracted” epidemiological transitions proposed by Frenk [5] based on experiences in Mexico. The epidemiological transition in the Agincourt population began a reversal in the early 1990s [6, 18] that continued until around 2004–2007. This reversal was driven mostly by increases in mortality attributable to HIV/AIDS and TB. Only in recent years has the transition reversed again and started to move in the positive direction, with falling overall mortality and standard (as predicted by the classic theory of the epidemiological transition) changes to the cause of death distribution. This results from the widespread availability and uptake of antiretroviral treatment (ART) that has successfully reduced the number of deaths attributable to HIV/AIDS and TB. Provision of ART started in three district hospitals surrounding the study area between 2004 and 2005 [28, 47]. In 2007 a private community health centre specializing in HIV care and treatment services and run in partnership with the Department of Health, the Bhubezi Community Health Centre, started operating in the study area [47]. Provision of ART thereafter extended to public primary care clinics in the study area between 2008 and 2009 and has become widespread since 2010. However, despite improvements in recent years and overall mortality in children under the age of five years reaching the levels of 20 years before, largely due to the success of prevention of mother-to-child transmission (PMTCT) programmes [48], in most age groups indicators of the epidemiological transition have yet to reach the levels they occupied in the early 1990s. Thus the epidemiological transition is still evolving, having been significantly delayed by the HIV/AIDS epidemic. The progress of the transition has also been characterized by persistent gender differences with faster positive progression in females than males. Similar to other southern African settings, this may be because rates of HIV testing and linkage to and retention in care are higher in females than in males [49–52].

We acknowledge several limitations to this study. First, since updates of vital events in the Agincourt HDSS occur once a year there is a possibility that some still births, neonatal and infant deaths may not be recorded particularly when births and deaths occur between consecutive household visits [32]. However, this bias is minimal in recent years because since 2000 names of the most recent child born to each woman appear on the pre-populated household roster and since 2006 there is careful probing for pregnancies and births since the last recorded child by asking about pregnancy status of every woman of childbearing [32]. Second, we used data from one defined geographic region in rural South Africa. As such, the applicability of our findings elsewhere may not be easy to establish. However, similar to another earlier study [14], this study provides clear evidence of the major interruption to the classical epidemiological transition brought about by the HIV/AIDS epidemic. Second, while this study’s goal was to characterize mortality patterns over time and empirically assess the changing relations between overall mortality levels and cause compositions, focusing on population-level patterns may mask heterogeneity in these patterns by social, economic and other indicators. Future analyses exploring heterogeneity in transition trajectories by social groups may identify important differentials and disparities as well as potential explanations of underlying patterns and drivers of epidemiological change.

Evidence suggests that the Agincourt population is undergoing dynamic socioeconomic change [34] while concurrently experiencing high prevalence of HIV [53] and risk factors for cardiometabolic diseases, particularly hypertension [54]. Our findings imply that the epidemiological transition will continue to be protracted in the near future, especially in the middle adult age categories. As more people living with HIV/AIDS access antiretroviral treatment, concentration of mortality will shift towards older age categories and the contribution of cardiovascular and other chronic non-communicable diseases will become more apparent. Further, while baseline data suggests little interaction of ART and cardiometabolic disease risk [54], greater ART uptake and resulting prolonged survival highlights the need for further studies on the interaction of HIV, cardiometabolic disease and ageing. Hence, our results suggest a need to realign the health care system to cater concurrently for multiple disease conditions.

## Conclusion

This study has provided a detailed examination of the changing epidemiological profile of a rural South African population prior to and throughout the emergence of the HIV/AIDS epidemic in the absence of treatment, and the resulting changes in the context of PMTCT and ART rollout. Grounded in a robust statistical framework permitting detailed empirical assessment relating mortality levels to cause of death composition, our findings suggest that the Agincourt population is experiencing a protracted transition, with multiple stages overlapping and changes incomplete. This calls for continuous monitoring of the trajectory of the transition in order to advise policy makers around health planning and resource allocation and highlights the value of HDSS. Increasingly, the intersection and interaction of HIV and ART, non-communicable disease risk factors such as rising hypertension, obesity and type-2 diabetes and complex social, economic and behavioral changes occurring in the population (for example, rising labour migration in young women [55]) will impact continued progress in reducing premature mortality and improving health. This study highlights the need for integrated healthcare planning and program delivery to improve access and adherence to treatment for HIV and non-communicable diseases. Finally, our findings from a local, rural setting over an extended period contribute to the evidence base to inform further refinement and advancement of health and epidemiological transition theory.

## Additional file

Additional file 1: Table S1. Multinomial logistic regression of death by cause, sex, age, and time period. (DOCX 51 kb)
