# Supplementary material for: Progression of the epidemiological transition in a rural South African setting: findings from population surveillance in Agincourt, 1993–2013
Source: BMC Public Health. 2017 May 10;17:424. doi: 10.1186/s12889-017-4312-x (PMC5424387; doi:10.1186/s12889-017-4312-x)
Supplement: Additional file 1: Table S1. — Multinomial logistic regression of death by cause, sex, age, and time period. (DOCX 51 kb) [file 12889_2017_4312_MOESM1_ESM.docx]

**Additional file 1: Table 1.** Multinomial logistic regression of death by cause, sex, age, and time period

| Variable | Relative Risk Ratio | 95% CI | p-value |
| --- | --- | --- | --- |
| **HIV/TB** |  |  |  |
| *Sex* |  |  |  |
| Female | 1.00 | – | – |
| Male | 1.10 | [0.86,1.40] | 0.442 |
| *Age Groups* |  |  |  |
| 0-4 | 1.00 | – | – |
| 5-14 | 0.09 | [0.05,0.16] | < 0.001 |
| 15-49 | 0.78 | [0.59,1.05] | 0.102 |
| 50-64 | 1.81 | [1.28,2.56] | < 0.001 |
| 65+ | 3.47 | [2.51,4.81] | < 0.001 |
| *Time Period* |  |  |  |
| 1993-1997 | 1.00 | – | – |
| 1998–2000 | 2.00 | [1.41,2.83] | < 0.001 |
| 2001–2003 | 3.66 | [2.69,5.00] | < 0.001 |
| 2004–2007 | 2.78 | [2.05,3.78] | < 0.001 |
| 2008–2010 | 1.44 | [1.02,2.04] | 0.040 |
| 2011–2013 | 0.78 | [0.51,1.19] | 0.249 |
| *Interactions between Sex and Age* | |  |  |
| Male ***X*** age 5–14 | 1.13 | [0.75,1.71] | 0.562 |
| Male ***X*** age 15–49 | 0.92 | [0.76,1.11] | 0.372 |
| Male ***X*** age 50–64 | 1.92 | [1.53,2.41] | < 0.001 |
| Male ***X*** age 65+ | 2.72 | [2.15,3.44] | < 0.001 |
| *Interactions between Sex and Time* | |  |  |
| Male ***X*** 1998–2000 | 0.94 | [0.72,1.22] | 0.644 |
| Male ***X*** 2001–2003 | 0.86 | [0.68,1.08] | 0.202 |
| Male ***X*** 2004–2007 | 0.84 | [0.67,1.04] | 0.107 |
| Male ***X*** 2008–2010 | 0.98 | [0.78,1.24] | 0.877 |
| Male ***X*** 2011–2013 | 0.88 | [0.68,1.13] | 0.298 |
| *Interactions between Age and Time* | |  |  |
| Age 5-14 ***X*** 1998–2000 | 0.39 | [0.14,1.08] | 0.069 |
| Age 5-14 ***X*** 2001–2003 | 0.77 | [0.36,1.63] | 0.493 |
| Age 5-14 ***X*** 2004–2007 | 1.11 | [0.54,2.27] | 0.778 |
| Age 5-14 ***X*** 2008–2010 | 2.80 | [1.36,5.78] | 0.005 |
| Age 5-14 ***X*** 2011–2013 | 1.86 | [0.76,4.55] | 0.177 |
| Age 15-49 ***X*** 1998–2000 | 1.14 | [0.79,1.66] | 0.485 |
| Age 15-49 ***X*** 2001–2003 | 1.45 | [1.04,2.02] | 0.029 |
| Age 15-49 ***X*** 2004–2007 | 2.23 | [1.61,3.10] | < 0.001 |
| Age 15-49 ***X*** 2008–2010 | 2.72 | [1.88,3.94] | < 0.001 |
| Age 15-49 ***X*** 2011–2013 | 2.87 | [1.85,4.45] | < 0.001 |
| Age 50-64 ***X*** 1998–2000 | 0.51 | [0.32,0.84] | 0.008 |
| Age 50-64 ***X*** 2001–2003 | 0.79 | [0.53,1.19] | 0.260 |
| Age 50-64 ***X*** 2004–2007 | 1.45 | [0.98,2.13] | 0.062 |
| Age 50-64 ***X*** 2008–2010 | 1.72 | [1.12,2.65] | 0.014 |
| Age 50-64 ***X*** 2011–2013 | 1.94 | [1.18,3.21] | 0.010 |
| Age 65+ ***X*** 1998–2000 | 0.62 | [0.41,0.96] | 0.032 |
| Age 65+ ***X*** 2001–2003 | 0.41 | [0.28,0.61] | < 0.001 |
| Age 65+ ***X*** 2004–2007 | 0.54 | [0.37,0.78] | 0.001 |
| Age 65+ ***X*** 2008–2010 | 0.91 | [0.60,1.38] | 0.649 |
| Age 65+ ***X*** 2011–2013 | 1.32 | [0.81,2.15] | 0.269 |
| **Other Communicable Causes** | | |  |
| *Sex* |  |  |  |
| Female | 1.00 | – | – |
| Male | 0.90 | [0.69,1.16] | 0.408 |
| *Age Groups* |  |  |  |
| 0-4 | 1.00 | – | – |
| 5-14 | 0.06 | [0.04,0.11] | < 0.001 |
| 15-49 | 0.11 | [0.07,0.15] | < 0.001 |
| 50-64 | 0.21 | [0.12,0.36] | < 0.001 |
| 65+ | 1.01 | [0.70,1.44] | 0.975 |
| *Time Period* |  |  |  |
| 1993-1997 | 1.00 | – | – |
| 1998–2000 | 1.40 | [1.03,1.91] | 0.033 |
| 2001–2003 | 1.84 | [1.37,2.46] | < 0.001 |
| 2004–2007 | 2.04 | [1.57,2.65] | < 0.001 |
| 2008–2010 | 1.89 | [1.45,2.47] | < 0.001 |
| 2011–2013 | 1.36 | [1.03,1.81] | 0.032 |
| *Interactions between Sex and Age* | |  |  |
| Male ***X*** age 5–14 | 1.08 | [0.70,1.65] | 0.734 |
| Male ***X*** age 15–49 | 0.80 | [0.64,0.99] | 0.038 |
| Male ***X*** age 50–64 | 2.05 | [1.46,2.89] | < 0.001 |
| Male ***X*** age 65+ | 1.64 | [1.25,2.17] | < 0.001 |
| *Interactions between Sex and Time* | |  |  |
| Male ***X*** 1998–2000 | 1.09 | [0.75,1.59] | 0.638 |
| Male ***X*** 2001–2003 | 1.16 | [0.82,1.65] | 0.402 |
| Male ***X*** 2004–2007 | 1.32 | [0.97,1.80] | 0.078 |
| Male ***X*** 2008–2010 | 1.48 | [1.09,2.01] | 0.013 |
| Male ***X*** 2011–2013 | 1.10 | [0.80,1.51] | 0.551 |
| *Interactions between Age and Time* | |  |  |
| Age 5-14 ***X*** 1998–2000 | 0.72 | [0.32,1.60] | 0.418 |
| Age 5-14 ***X*** 2001–2003 | 0.45 | [0.19,1.05] | 0.063 |
| Age 5-14 ***X*** 2004–2007 | 0.62 | [0.31,1.23] | 0.168 |
| Age 5-14 ***X*** 2008–2010 | 1.02 | [0.53,1.97] | 0.944 |
| Age 5-14 ***X*** 2011–2013 | 0.79 | [0.36,1.71] | 0.547 |
| Age 15-49 ***X*** 1998–2000 | 0.92 | [0.55,1.53] | 0.738 |
| Age 15-49 ***X*** 2001–2003 | 0.93 | [0.58,1.48] | 0.747 |
| Age 15-49 ***X*** 2004–2007 | 1.19 | [0.79,1.79] | 0.409 |
| Age 15-49 ***X*** 2008–2010 | 1.59 | [1.05,2.38] | 0.027 |
| Age 15-49 ***X*** 2011–2013 | 2.88 | [1.90,4.36] | < 0.001 |
| Age 50-64 ***X*** 1998–2000 | 0.78 | [0.36,1.73] | 0.546 |
| Age 50-64 ***X*** 2001–2003 | 0.81 | [0.40,1.65] | 0.562 |
| Age 50-64 ***X*** 2004–2007 | 0.77 | [0.40,1.47] | 0.429 |
| Age 50-64 ***X*** 2008–2010 | 1.23 | [0.66,2.28] | 0.517 |
| Age 50-64 ***X*** 2011–2013 | 2.31 | [1.26,4.26] | 0.007 |
| Age 65+ ***X*** 1998–2000 | 0.67 | [0.40,1.14] | 0.144 |
| Age 65+ ***X*** 2001–2003 | 0.39 | [0.22,0.67] | 0.001 |
| Age 65+ ***X*** 2004–2007 | 0.38 | [0.24,0.61] | < 0.001 |
| Age 65+ ***X*** 2008–2010 | 0.57 | [0.36,0.90] | 0.017 |
| Age 65+ ***X*** 2011–2013 | 1.32 | [0.85,2.06] | 0.212 |
| **Non-communicable Causes** | | |  |
| *Sex* |  |  |  |
| Female | 1.00 | – | – |
| Male | 1.37 | [0.88,2.12] | 0.159 |
| *Age Groups* |  |  |  |
| 0-4 | 1.00 | – | – |
| 5-14 | 0.48 | [0.20,1.16] | 0.104 |
| 15-49 | 2.22 | [1.20,4.11] | 0.011 |
| 50-64 | 15.05 | [8.12,27.90] | < 0.001 |
| 65+ | 76.85 | [42.68,138.36] | < 0.001 |
| *Time Period* |  |  |  |
| 1993-1997 | 1.00 | – | – |
| 1998–2000 | 1.78 | [0.82,3.84] | 0.144 |
| 2001–2003 | 2.47 | [1.22,5.00] | 0.012 |
| 2004–2007 | 1.58 | [0.78,3.23] | 0.205 |
| 2008–2010 | 1.93 | [0.95,3.92] | 0.071 |
| 2011–2013 | 1.57 | [0.75,3.29] | 0.234 |
| *Interactions between Sex and Age* | |  |  |
| Male ***X*** age 5–14 | 0.74 | [0.36,1.52] | 0.406 |
| Male ***X*** age 15–49 | 0.89 | [0.58,1.36] | 0.590 |
| Male ***X*** age 50–64 | 1.09 | [0.71,1.67] | 0.707 |
| Male ***X*** age 65+ | 1.05 | [0.69,1.59] | 0.818 |
| *Interactions between Sex and Time* | |  |  |
| Male ***X*** 1998–2000 | 0.93 | [0.71,1.22] | 0.592 |
| Male ***X*** 2001–2003 | 1.09 | [0.84,1.41] | 0.513 |
| Male ***X*** 2004–2007 | 1.22 | [0.97,1.53] | 0.093 |
| Male ***X*** 2008–2010 | 0.99 | [0.79,1.25] | 0.934 |
| Male ***X*** 2011–2013 | 1.02 | [0.81,1.28] | 0.872 |
| *Interactions between Age and Time* | |  |  |
| Age 5-14 ***X*** 1998–2000 | 0.43 | [0.12,1.58] | 0.205 |
| Age 5-14 ***X*** 2001–2003 | 0.24 | [0.06,0.91] | 0.035 |
| Age 5-14 ***X*** 2004–2007 | 0.74 | [0.25,2.20] | 0.590 |
| Age 5-14 ***X*** 2008–2010 | 0.54 | [0.17,1.75] | 0.304 |
| Age 5-14 ***X*** 2011–2013 | 0.46 | [0.13,1.65] | 0.233 |
| Age 15-49 ***X*** 1998–2000 | 0.94 | [0.42,2.10] | 0.877 |
| Age 15-49 ***X*** 2001–2003 | 0.78 | [0.37,1.63] | 0.507 |
| Age 15-49 ***X*** 2004–2007 | 1.40 | [0.67,2.94] | 0.368 |
| Age 15-49 ***X*** 2008–2010 | 1.31 | [0.63,2.76] | 0.469 |
| Age 15-49 ***X*** 2011–2013 | 1.81 | [0.84,3.90] | 0.130 |
| Age 50-64 ***X*** 1998–2000 | 0.72 | [0.32,1.63] | 0.427 |
| Age 50-64 ***X*** 2001–2003 | 0.70 | [0.33,1.47] | 0.344 |
| Age 50-64 ***X*** 2004–2007 | 1.18 | [0.56,2.49] | 0.659 |
| Age 50-64 ***X*** 2008–2010 | 1.03 | [0.49,2.19] | 0.929 |
| Age 50-64 ***X*** 2011–2013 | 1.17 | [0.54,2.54] | 0.691 |
| Age 65+ ***X*** 1998–2000 | 0.63 | [0.29,1.37] | 0.240 |
| Age 65+ ***X*** 2001–2003 | 0.45 | [0.22,0.92] | 0.028 |
| Age 65+ ***X*** 2004–2007 | 0.78 | [0.38,1.60] | 0.499 |
| Age 65+ ***X*** 2008–2010 | 0.85 | [0.41,1.73] | 0.646 |
| Age 65+ ***X*** 2011–2013 | 1.05 | [0.50,2.22] | 0.888 |
| **Injuries** |  |  |  |
| *Sex* | 1.00 | – | – |
| Female | 0.80 | [0.42,1.51] | 0.486 |
| Male |  |  |  |
| *Age Groups* |  |  |  |
| 0-4 | 1.00 | – | – |
| 5-14 | 0.37 | [0.15,0.89] | 0.026 |
| 15-49 | 1.09 | [0.55,2.14] | 0.804 |
| 50-64 | 2.46 | [1.11,5.44] | 0.026 |
| 65+ | 2.15 | [0.89,5.16] | 0.087 |
| *Time Period* |  |  |  |
| 1993-1997 | 1.00 | – | – |
| 1998–2000 | 1.1 | [0.42,2.87] | 0.851 |
| 2001–2003 | 0.77 | [0.26,2.24] | 0.630 |
| 2004–2007 | 0.65 | [0.24,1.77] | 0.395 |
| 2008–2010 | 0.7 | [0.26,1.93] | 0.493 |
| 2011–2013 | 1.18 | [0.50,2.79] | 0.699 |
| *Interactions between Sex and Age* | |  |  |
| Male ***X*** age 5–14 | 2.98 | [1.37,6.47] | 0.006 |
| Male ***X*** age 15–49 | 5.13 | [2.79,9.43] | < 0.001 |
| Male ***X*** age 50–64 | 4.03 | [1.97,8.21] | < 0.001 |
| Male ***X*** age 65+ | 4.17 | [1.97,8.83] | < 0.001 |
| *Interactions between Sex and Time* | |  |  |
| Male ***X*** 1998–2000 | 0.94 | [0.55,1.63] | 0.835 |
| Male ***X*** 2001–2003 | 1.07 | [0.65,1.78] | 0.780 |
| Male ***X*** 2004–2007 | 1.16 | [0.72,1.87] | 0.539 |
| Male ***X*** 2008–2010 | 1.13 | [0.67,1.93] | 0.642 |
| Male ***X*** 2011–2013 | 1.33 | [0.80,2.21] | 0.273 |
| *Interactions between Age and Time* | |  |  |
| Age 5-14 ***X*** 1998–2000 | 1.06 | [0.31,3.58] | 0.923 |
| Age 5-14 ***X*** 2001–2003 | 2.09 | [0.59,7.35] | 0.252 |
| Age 5-14 ***X*** 2004–2007 | 1.36 | [0.40,4.71] | 0.623 |
| Age 5-14 ***X*** 2008–2010 | 0.80 | [0.20,3.11] | 0.742 |
| Age 5-14 ***X*** 2011–2013 | 0.69 | [0.22,2.16] | 0.519 |
| Age 15-49 ***X*** 1998–2000 | 0.87 | [0.32,2.35] | 0.780 |
| Age 15-49 ***X*** 2001–2003 | 1.60 | [0.54,4.77] | 0.397 |
| Age 15-49 ***X*** 2004–2007 | 1.79 | [0.64,4.97] | 0.266 |
| Age 15-49 ***X*** 2008–2010 | 1.24 | [0.44,3.49] | 0.677 |
| Age 15-49 ***X*** 2011–2013 | 0.71 | [0.30,1.71] | 0.446 |
| Age 50-64 ***X*** 1998–2000 | 0.81 | [0.26,2.55] | 0.717 |
| Age 50-64 ***X*** 2001–2003 | 1.27 | [0.38,4.26] | 0.704 |
| Age 50-64 ***X*** 2004–2007 | 1.02 | [0.32,3.25] | 0.974 |
| Age 50-64 ***X*** 2008–2010 | 0.90 | [0.28,2.91] | 0.854 |
| Age 50-64 ***X*** 2011–2013 | 0.57 | [0.21,1.57] | 0.276 |
| Age 65+ ***X*** 1998–2000 | 0.95 | [0.27,3.34] | 0.935 |
| Age 65+ ***X*** 2001–2003 | 2.05 | [0.57,7.34] | 0.271 |
| Age 65+ ***X*** 2004–2007 | 1.96 | [0.59,6.52] | 0.274 |
| Age 65+ ***X*** 2008–2010 | 0.91 | [0.24,3.37] | 0.882 |
| Age 65+ ***X*** 2011–2013 | 0.53 | [0.16,1.73] | 0.295 |
| **Indeterminate** |  |  |  |
| *Sex* |  |  |  |
| Female | 1.00 | – | – |
| Male | 1.13 | [0.83,1.53] | 0.434 |
| *Age Groups* |  |  |  |
| 0-4 | 1.00 | – | – |
| 5-14 | 0.09 | [0.05,0.19] | < 0.001 |
| 15-49 | 0.29 | [0.20,0.42] | < 0.001 |
| 50-64 | 1.56 | [1.03,2.35] | 0.036 |
| 65+ | 5.89 | [4.17,8.34] | < 0.001 |
| *Time Period* |  |  |  |
| 1993-1997 | 1.00 | – | – |
| 1998–2000 | 1.76 | [1.18,2.63] | 0.006 |
| 2001–2003 | 1.49 | [0.99,2.25] | 0.057 |
| 2004–2007 | 1.46 | [1.00,2.13] | 0.048 |
| 2008–2010 | 0.68 | [0.43,1.10] | 0.116 |
| 2011–2013 | 0.78 | [0.49,1.25] | 0.307 |
| *Interactions between Sex and Age* | |  |  |
| Male ***X*** age 5–14 | 0.97 | [0.57,1.65] | 0.909 |
| Male ***X*** age 15–49 | 1.97 | [1.48,2.61] | < 0.001 |
| Male ***X*** age 50–64 | 1.84 | [1.31,2.60] | < 0.001 |
| Male ***X*** age 65+ | 1.27 | [0.95,1.71] | 0.111 |
| *Interactions between Sex and Time* | |  |  |
| Male ***X*** 1998–2000 | 0.86 | [0.61,1.22] | 0.392 |
| Male ***X*** 2001–2003 | 1.07 | [0.77,1.49] | 0.685 |
| Male ***X*** 2004–2007 | 1.07 | [0.80,1.44] | 0.649 |
| Male ***X*** 2008–2010 | 1.13 | [0.79,1.62] | 0.494 |
| Male ***X*** 2011–2013 | 0.93 | [0.63,1.36] | 0.693 |
| *Interactions between Age and Time* | |  |  |
| Age 5-14 ***X*** 1998–2000 | 0.72 | [0.27,1.93] | 0.520 |
| Age 5-14 ***X*** 2001–2003 | 0.89 | [0.34,2.32] | 0.818 |
| Age 5-14 ***X*** 2004–2007 | 1.87 | [0.85,4.13] | 0.120 |
| Age 5-14 ***X*** 2008–2010 | 1.01 | [0.32,3.16] | 0.987 |
| Age 5-14 ***X*** 2011–2013 | 1.93 | [0.74,5.02] | 0.179 |
| Age 15-49 ***X*** 1998–2000 | 0.76 | [0.47,1.24] | 0.273 |
| Age 15-49 ***X*** 2001–2003 | 1.18 | [0.74,1.88] | 0.487 |
| Age 15-49 ***X*** 2004–2007 | 1.40 | [0.91,2.15] | 0.126 |
| Age 15-49 ***X*** 2008–2010 | 1.70 | [1.01,2.86] | 0.046 |
| Age 15-49 ***X*** 2011–2013 | 1.07 | [0.62,1.82] | 0.815 |
| Age 50-64 ***X*** 1998–2000 | 0.49 | [0.27,0.88] | 0.017 |
| Age 50-64 ***X*** 2001–2003 | 0.72 | [0.41,1.25] | 0.238 |
| Age 50-64 ***X*** 2004–2007 | 1.03 | [0.63,1.67] | 0.912 |
| Age 50-64 ***X*** 2008–2010 | 0.97 | [0.53,1.79] | 0.927 |
| Age 50-64 ***X*** 2011–2013 | 0.50 | [0.25,0.98] | 0.045 |
| Age 65+ ***X*** 1998–2000 | 0.62 | [0.38,0.99] | 0.044 |
| Age 65+ ***X*** 2001–2003 | 0.65 | [0.40,1.04] | 0.075 |
| Age 65+ ***X*** 2004–2007 | 0.53 | [0.34,0.83] | 0.005 |
| Age 65+ ***X*** 2008–2010 | 0.73 | [0.42,1.27] | 0.266 |
| Age 65+ ***X*** 2011–2013 | 0.63 | [0.36,1.10] | 0.104 |
